# Reconstruction of radial deviation after preaxial polydactyly surgery: use of an ulnar fascial flap

**DOI:** 10.3389/fped.2024.1433249

**Published:** 2024-11-18

**Authors:** Hua Chen, Kang Wu, Hongrui Wang, Yong Hu, Yang Wang

**Affiliations:** Department of Hand and Foot Surgery, The Second Hospital of Shandong University, Jinan, Shandong, China

**Keywords:** preaxial polydactyly, secondary deformity, Wassel, congenital hand anomaly, fascial flap

## Abstract

**Background:**

Radial deviation of the interphalangeal (IP) joint is a common complication of treating Wassel type IV-D preaxial polydactyly. Long-term radial deviation can place excessive tension on the radial skin and cause overabundance of the ulnar skin. To overcome this problem, we aimed to utilize a fascial flap for ulnar reconstruction of a defect in the radial skin.

**Methods:**

We used a fascial flap for ulnar reconstruction of a defect in the radial skin, eight patients (average: 12 years, range: 5–33 years) who underwent reconstructive surgery at our department for radial deviation of the interphalangeal (IP) joint after the initial operation were included between August 2017 and August 2021. An incision was made on the radial side of the thumb. An olive-shaped flap was designed on the ulnar eminence of the IP joint. The skin and fascia in the other three quadrants were incised. While the flap was protected, children with epiphyseal plates underwent wedge osteotomy at the maximum ulnar deviation of the proximal phalanx, and adults underwent direct fusion of the distal IP joint. Absorbable sutures were used to suture the flap. The Tada functional and visual analog scale (VAS) scores were recorded before and after the operation.

**Results:**

All flaps survived without infection or necrosis. The preoperative and postoperative Tada scores were 1 and 5, respectively, and the preoperative and postoperative VAS scores were 3 and 9, respectively. The postoperative scores showed a statistically significant degree of improvement.

**Conclusions:**

An ulnar fascial flap is an effective and feasible option for repair of radial deviation following surgery for preaxial polydactyly.

## Introduction

Preaxial polydactyly is a common congenital malformation of the upper extremities. The exact cause of this anomaly is unknown, but genetic mutations during the development of an embryo are considered to be the probable cause. Preaxial or thumb polydactyly occurs on the radial aspect of the limb or anatomic lateral aspect of the extremity. The main phenotype of this congenital difference is the presence of an extra thumb. This deformity occurs in 1 in 1,000–10,000 newborns. The Wassel classification system is used for classifying different types of preaxial polydactyly, and Wassel type IV-D is the most complex type (1–3). Surgical methods for treating this anomaly are categorized into the following two groups: resection of one of the duplicated thumbs with reconstruction of the other thumb, and the Bilhaut-Cloquet procedure (3–5).

Radial deviation of the interphalangeal (IP) joint is a common complication following surgical repair of this disorder. Many reconstruction methods, including osteotomy/arthrodesis, tendon relocation, and joint capsule tightening (6–10), have been published. Radial deviation of the IP joint can be associated with considerable skin defects. This poses a perplexing problem for hand and plastic surgeons. A few reports have discussed the methods to repair defects in the radial skin. The most common technique is the application of a Z-shaped local flap. Long-term radial deviation and scars from the initial operation can place excessive tension on the radial skin; however, the Z-shaped flap is not large enough to cover the defect in the radial skin with correction (6). The skin over the ulnar area is expanded, and the subcutaneous fascial vascular network on the dorsal skin of the thumb allows an ulnar local fascial flap (11, 12). Herein, we describe our technique for reconstruction of radial deviation of the IP joint after surgery for preaxial polydactyly, which involves the use of an ulnar local fascial flap to cover the defect in the radial skin and tendon relocation and osteotomy/arthrodesis to correct the deviation of the IP joint of the reconstructed thumb.

## Methods

### Ethics approval and consent

The study protocol was approved by the Research Ethics Committee of the Second Hospital of Shandong University. Informed consent was obtained from all patients or their parents.

### Patients and clinical characteristics

Between August 2017 and August 2022, eight patients, two females and six males, who presented with radial deviation of the IP joint underwent surgery at the hand/foot surgery department (Table 1). The mean age at surgery was 12 years (range 5–33 years). In four cases, the right thumb was affected, and in the other four cases, the left thumb was affected. Two patients presented with ulnar deviation of the metacarpophalangeal joint and partial residual proximal phalangeal base in the radial area. These deformities were not completely resected in the initial operation. All patients were treated with an ulnar fascial flap to cover the defect in the radial skin of the IP joint.

**Table 1:** Patient data and the preoperative/final follow-up scores.

Patient	Sex	Age at the operation (months)	Laterality	Tada (preoperative)	VAS (preoperative)	Arthrodesis performed	Follow-up evaluation (months)	Tada (follow-up)	VAS (follow-up)
1	M	52	L	1	4	No	48	4	8
2	F	72	L	1	3	No	41	6	10
3	M	168	L	1	3	No	12	4	7
4	M	108	R	1	3	No	12	6	10
5	M	99	R	1	4	No	7	6	9
6	M	198	R	2	3	No	6	6	9
7	M	75	L	1	4	No	6	6	10
8	F	394	R	1	3	Yes	4	5	9

### Surgical technique

**Figure 1:**
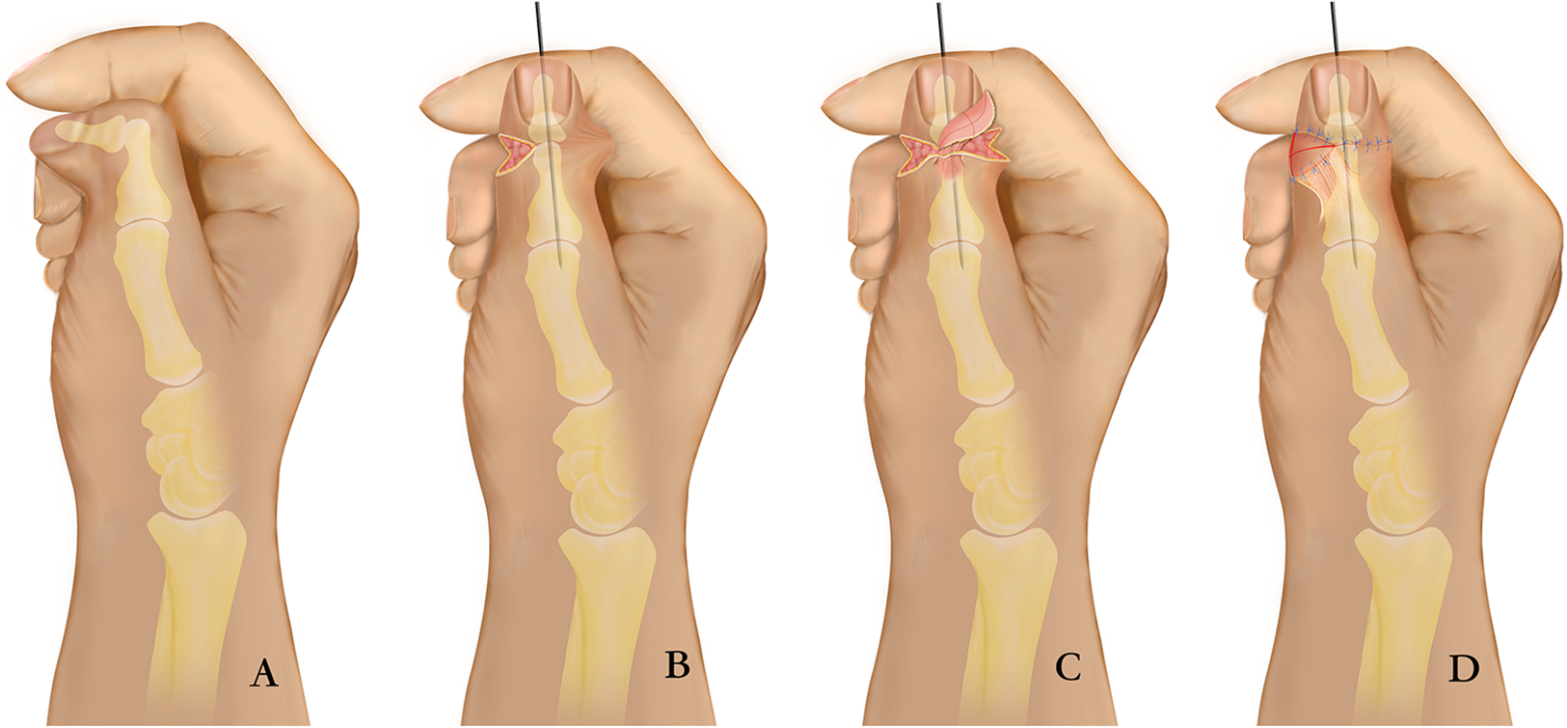
Diagram showing the surgical technique. **(A)** Preoperative dorsal view; **(B)** The deviated IP joint was corrected, and children with epiphyseal plates underwent wedge osteotomy at the maximum ulnar deviation of the proximal phalanx (in adults, the distal IP joint was directly fused); **(C)** Flap harvest and transfer; **(D)** Postoperative dorsal view.

**Figure 2:**
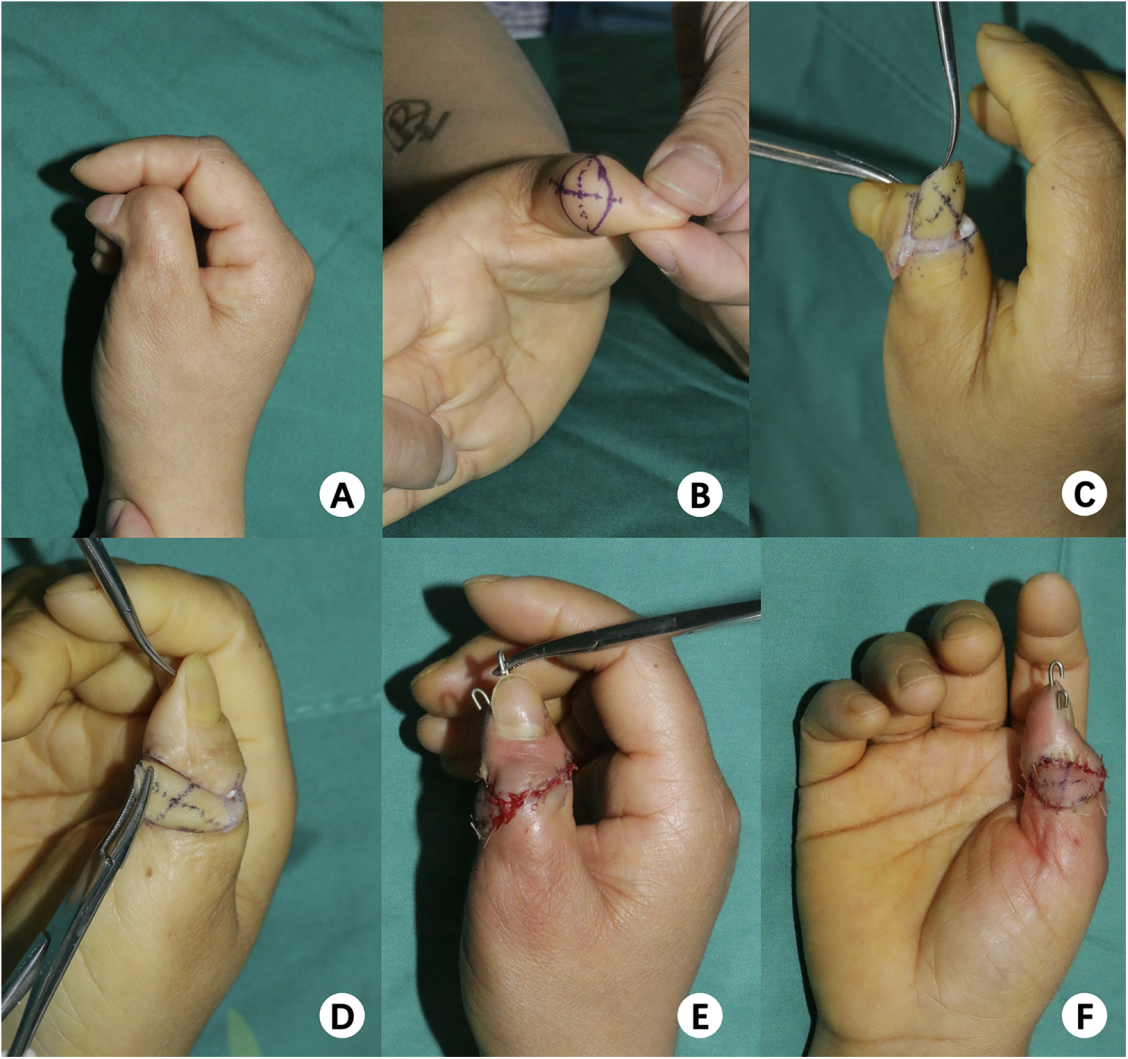
Representative case of a 33-year-old female with deviation of the right thumb. **(A)** Preoperative dorsal view, **(B)** showing design of flap, **(C,D)** Intraoperative, flap harvested transferred covered, **(E,F)** Immediate postoperative result: dorsal lateral view.

All patients underwent surgery under general anesthesia. Tourniquets were used to prevent bleeding. The surgeon made an incision on the radial side of the thumb. The distal end of the incision reached the insertion point of the flexor and extensor tendons, and the proximal end of the incision reached the middle proximal phalanx; in two cases of metacarpophalangeal joint deviation, the incision extended up to the metacarpophalangeal joint. After making the skin incision, the surgical team was unable to locate the radial neurovascular bundle in seven cases. After resection of the scar tissue, the extensor and flexor tendons were isolated, and the team confirmed that there was no interlacing tendinous tissue between these two tendons (6, 13). The surgical team took utmost care to avoid crossing the dorsal midline of the thumb during the release of the soft tissue in order to prevent damage to the vascular network beneath the fascia. The surgical team released the capsule on the radial side of the IP joint. The deviated IP joint was corrected manually (Figure 1). The skin was incised through the coronal axis of the thumb perpendicular to the site on the radial area with the highest tension, without crossing the palmar and dorsal midlines of the thumb. After complete release of the tense radial scar, the area of the defect was measured. An olive-shaped flap, which had the same area as the radial defect, was designed on the ulnar eminence of the IP joint. The longitudinal axis of the flap was perpendicular to the sagittal axis of the thumb. The flap was divided into four quadrants; the pedicle was located in the fourth quadrant of the dorsal proximal part, and the skin and fascia in the other three quadrants were incised. The pedicle was isolated by using 2.5-fold magnifying head-mounted loupes. The skin was incised with micro scissors to protect the fascial tissue and capillary network connected to the flap. The width of the pedicle of the fascial tissue was equal to that of the fourth quadrant of the flap. First, the superficial layer of the pedicle fascia was isolated, and then the deep layer of the pedicle fascia was isolated. Synovial tissues of the extensor tendon were protected; the pedicle of the flap was set oblique to the radial side of the thumb. The length and width of the flap were measured so that it could easily cover the radial defect, and the surgical team generally did not cross the dorsal midline of the thumb. It was difficult to distinguish the fascial vascular network with the naked eye, and unnamed vessels were detected. We suggest that even experienced microsurgeons operate under magnification when separating the pedicle to avoid damaging the vascular network. Under ordinary circumstances, if the width and thickness of the pedicle were adequate, the blood supply of the flap was adequate. While the flap was protected, children with open epiphyses underwent wedge osteotomy at the maximum ulnar deviation of the proximal phalanx (in adults, the distal IP joint was directly fused). In pediatric cases, tendons were reconstructed using the turn-page procedure, and the axis of the thumb was corrected (14). Internal fixation with a 1.0-mm/1.2-mm Kirschner wire, longitudinally or crosswise, sutured the flexor and extensor tendons to the ulnar base of the distal phalanx. Under fluoroscopy, the axis of the thumb was found to be satisfactory (Figure 2). The flap was inset with 6-0 absorbable sutures. The pedicle of the flap was not under tension and the defect area was small; thus, the flap was transferred through a subcutaneous tunnel. However, if the pedicle was under tension and the defect area was large, the flap was transferred through an open tunnel. An alcohol gauze bandage and a short arm plaster were applied. The dressing was changed every other day. x-ray images were taken 45 days after the operation, which confirmed osteotomy healing. Then, the Kirschner wire was removed to provide therapy. After 3–5 days, scar removal drugs and elastic bandages were used to compress the scars.

Postoperatively, daytime use of an elastic bandage on the finger helped shape the healing wound during the initial 3–6 month post operative period; with each bandage released hourly after 4–5 h to ensure circulation. At night, children's joints were immobilized with splints to prevent deviation. Passive exercises of the interphalangeal joints were generally discouraged, but active exercises such as pinching small objects were recommended to enhance mobility.

### Outcome measures

The Tada functional scoring system and the visual analog scale (VAS) assessment tools were administered before and after the surgery, and the preoperative and postoperative Tada and VAS scores were recorded (15).

### Statistical analysis

Statistical analysis was performed by the statistical software SPSS (IBM SPSS Statistics 21.0). The *t*-test was used for comparison, and *P* < 0.05 was considered statistically significant.

## Results

### Follow-up

The average follow-up period was 17 months (range, 4–48 months).

### Flap survival and patient satisfaction

**Figure 3:**
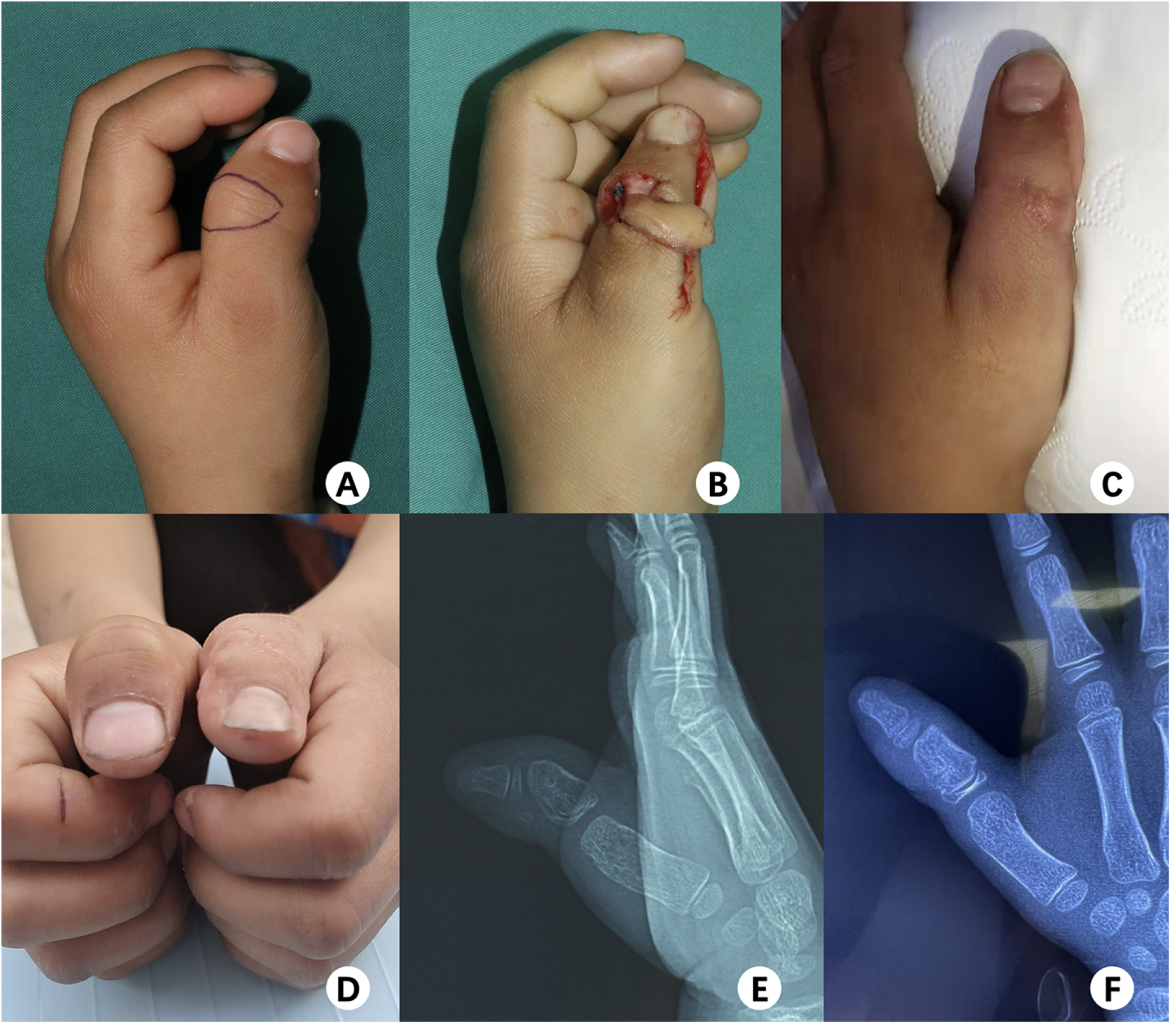
Seven-month follow-up following reconstruction in a 6-year-old boy with deviation of the left thumb. **(A)** Intraoperative, showing the design of the flap; **(B)** intraoperative, flap harvested; **(C,D)** good appearance and excellent function; **(E,F)** preoperative and postoperative x-rays, respectively.

All flaps survived without infection or necrosis. The patients and their guardians were satisfied with the contour, shape, and color of the flap (Figure 3).

### Results of outcome measures

The preoperative Tada score was 1, and the postoperative Tada score was 5, respectively. The preoperative VAS score was 3, and the postoperative VAS score was 9, respectively. Thus, postoperative scores showed a statistically significant improvement from baseline scores (*P* < 0.05) (Table 1).

## Discussion

Preaxial polydactyly is a common congenital deformity of the hand, and Wassel type IV-D is one of the most complex types (1–3). The abnormal anatomy of the Wassel type IV-D tendon and joint may be an important cause of the deviation of the IP joint after resection and reconstruction, which is generally inclined towards the radial side (3, 6, 16). In this study, deviation was the most common complaint of the patients and their guardians after the operation, and Tada and VAS scores were low. Reoperation was indicated in a wide variety of cases. Long-term deviation and scar contracture placed tension on the radial skin of the IP joint. Two issues tend to occur with reconstruction of the deviation. First, the defect created after release of the radial skin may not be covered by a simple procedure. Excess skin on the ulnar side is the second issue; it can influence the appearance, although it would resolve over time. Some authors have recommended a Z-shaped flap to resolve defects in the radial skin, but this flap is not suitable for large area defects and it cannot solve the problem of excess ulnar skin (8, 9, 14). The ulnar fascial flap used in this study for reconstruction of the skin defects associated with preaxial polydactyly offered the following four advantages: First, it could repair larger skin defects than Z-shaped flaps without affecting the blood supply of the thumb. Z-shaped flaps increase the length of skin by reducing the width; thus, leading to the formation of a constricting band. Further, blood supply of the thumb was poor, and local skin necrosis was observed in some cases. Second, defects in the radial skin were repaired, and the problem of excess ulnar skin was solved. After correcting the axial area of the deviating thumb, usually, there was excess ulnar skin of the IP joint, which created a visible contrast between the radial and ulnar areas. Although the axis of the thumb was normal, it was S-shaped in appearance, which had a negative cosmetic effect. Third, the color and quality of the flap were very similar to those of the defects because the flap was transferred from the same location on the ulnar side. Thus, it was very similar to the skin on the radial side. Finally, the osteotomy of phalanx and tendon relocation could be performed simultaneously via the same incision (6, 8). To correct the axis of the thumb, osteotomy and tendon relocation should be performed via the incision on the ulnar side of the thumb. Thus, no additional incisions are required.

However, the surgical procedure described in this study has some shortcomings. The blood supply of the fascial flap is derived from many small unnamed vessels. While harvesting the flap, the surgical team needs to pay great attention to the width and thickness of the fascial tissues. If the tension of transfer to the subcutaneous tunnel is very high, the flap should be transferred through an open tunnel, and the tension of the stitches between the flap and recipient bed should be relieved.

An ulnar local fascial flap is an effective and feasible treatment option for the repair of radial deviation after surgery for preaxial polydactyly.

## Data Availability

The raw data supporting the conclusions of this article will be made available by the authors, without undue reservation.
